# Silent atrial fibrillation in patients with an implantable cardioverter defibrillator and coronary artery disease (INDICO AF) trial: study rationale and design

**DOI:** 10.1007/s12471-018-1185-2

**Published:** 2018-10-24

**Authors:** S. W. E. Baalman, L. V. A. Boersma, C. P. Allaart, M. Meine, C. O. S. Scheerder, J. R. de Groot

**Affiliations:** 1 Department of Clinical and Experimental Cardiology, Heart Center, Amsterdam UMC, University of Amsterdam, Amsterdam, The Netherlands; 2 Department of Cardiology, Amsterdam UMC, VU University Medical Center, Amsterdam, The Netherlands; 3 Department of Cardiology, St. Antonius Hospital, Nieuwegein, The Netherlands; 4 Department of Cardiology, Division of Heart and Lungs, University Medical Center, Utrecht, The Netherlands; 5 Medtronic Bakken Research Center, Maastricht, The Netherlands

**Keywords:** Atrial fibrillation, Subclinical atrial fibrillation, Implantable cardioverter defibrillator, Remote monitoring

## Abstract

**Background:**

Timely detection of atrial fibrillation (AF) in implantable cardioverter defibrillator (ICD) patients is clinically important for prevention of AF-related complications and inappropriate shocks. Patients with coronary artery disease (CAD) and a dual or triple chamber ICD show a high incidence of device-detected AF. Whether CAD patients with a single chamber ICD carry a similar risk for device-detected AF remains unknown.

**Study design:**

The INDICO AF trial is an investigator-initiated, multicentre, observational study evaluating the incidence of subclinical AF (SCAF) in CAD patients who will receive a single chamber ICD as primary prevention for sudden cardiac death (SCD). Fifty patients will receive a single chamber ICD with an integrated RR interval based AF detection algorithm. In combination with remote monitoring, rhythm data will be collected monthly. The primary endpoint is the incidence of SCAF at 1 year of follow-up; secondary endpoints include time until 10 and 20% of the patients have a first episode of SCAF. All patients in whom SCAF is detected will be invited for an outpatient visit and will receive adequate anticoagulation treatment when appropriate according to the CHA2DS2-VASc score and current guidelines.

**Conclusion:**

The INDICO AF trial will quantify the incidence of SCAF in patients with an ICD and CAD. The study will underscore the clinical value of SCAF detection in single chamber ICD patients using remote patient monitoring and may improve patient care. This trial is registered at trialregister.nl with trial NTR6910.

## Background

The implantable cardioverter defibrillator (ICD) has shown to be successful in preventing sudden cardiac death (SCD) in patients with ischaemic and non-ischaemic cardiomyopathy, both as secondary or primary prevention. Currently, the European Society of Cardiology guidelines for the management of patients with ventricular arrhythmias and the prevention of SCD give a class IA recommendation for ICD implantation in patients with ischaemic cardiomyopathy with a reduced left ventricular ejection fraction (LVEF) ≤35% at least 6 weeks after myocardial infarction [1]. Besides protection against SCD, the monitoring capacities of the ICD may further improve patient care by timely detection of signs and determinants of deterioration.

Atrial fibrillation (AF) is the most common cardiac arrhythmia worldwide associated with a doubled mortality rate and a fivefold increase in ischaemic stroke even as independent risk factor [2]. Stroke prevention is therefore indicated in patients with an increased risk according to the CHA2DS2-VASc score [3]. The incidence and prevalence of AF are expected to further rise over the forthcoming years, partially due to ageing of the population and as a consequence of better survival of conditions that predispose to developing AF, such as heart failure and coronary artery disease (CAD). Indeed, the incidence of AF is considerably higher in patients with CAD and impaired left ventricular function.

Besides the increased risk of ischaemic stroke, AF in patients with ICDs has been associated with the occurrence of inappropriate shocks and is also a marker for worse prognosis [4]. Timely detection of AF in these patients is therefore of great clinical importance to prevent AF-associated complications.

The capability of conventional dual or triple chamber ICDs to detect subclinical AF (SCAF) has shown that the risk of SCAF in patients with CAD and a dual or triple chamber ICD is high [5]. It is likely that patients with CAD receiving a single chamber ICD for primary prevention of SCD carry a similarly high risk of SCAF, but so far, the incidence of SCAF in this patient population is unknown.

The newly developed and validated AF detection algorithm is incorporated into a single chamber ICD (VISIA AF™, Medtronic), and provides a platform to investigate incidence and prevalence of SCAF in single chamber ICD patients [6, 7]. This dedicated algorithm for AF detection reliably detects the presence or absence of AF. Detection of AF in patients with a reduced LVEF and CAD frequently has therapeutic implications, and delay of the diagnosis may negatively affect prognosis. The aim of the INDICO AF study, therefore, is to investigate the incidence of SCAF in a population of patients without AF, but with CAD and LVEF ≤35% undergoing ICD implantation for primary prevention of SCD.

## Study design

**Fig. 1:**
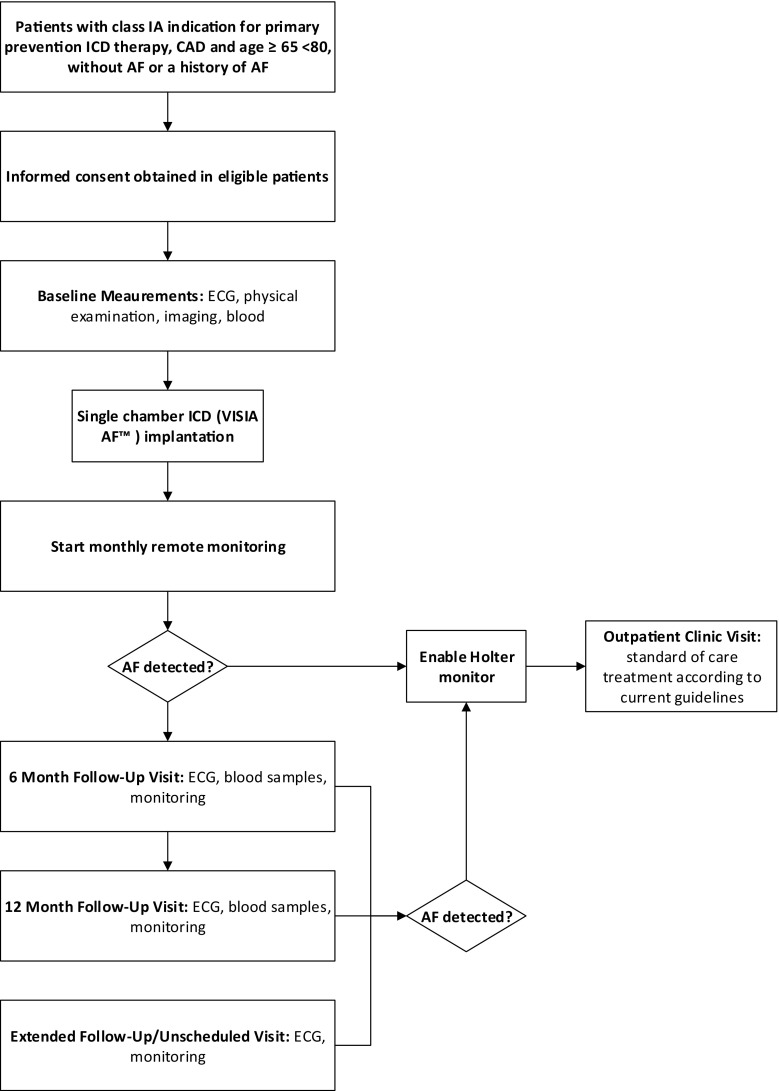
INDICO AF study flowchart. *ICD* implantable cardioverter-defibrillator, *CAD* coronary artery disease, *ECG* electrocardiogram, *AF* atrial fibrillation

The INDICO AF trial is a multicentre observational study, conducted within four high volume ICD implantation centres in the Netherlands. Fifty patients with CAD and LVEF ≤35% will receive a single chamber ICD including an algorithm-based rhythm recorder (VISIA AF™, Medtronic). The primary endpoint of the study is the incidence of SCAF after 1 year of follow-up; secondary endpoints include the time until 10 and 20% of the patients have a first episode of SCAF. In combination with remote monitoring, data will be collected monthly, and follow-up will continue until 20% of patients have had a first event of SCAF. In addition, blood will be drawn during the 6 and 12 month follow-up visits. All patients in whom SCAF is detected will be invited to the outpatient clinic and will receive standard of care treatment, according to the CHA2DS2-VASc score and current guidelines [3]. The trial organisation is shown in Fig. 1.

### Primary and secondary endpoints

The initial hypothesis of this study is that patients with CAD and a primary indication for a single chamber ICD are at high risk for SCAF. The primary endpoint is the incidence of SCAF in single chamber ICD patients with CAD at 1 year. If this is less than 20% after 1 year, we will continue the study until 20% of patients have had a first episode of SCAF. Further secondary endpoints include the burden of SCAF, the occurrence of transient ischaemic attack (TIA), stroke or systemic embolism and the incidence of appropriate or inappropriate ICD therapy. All events will be adjudicated by an independent event committee, whose members are not investigators in the INDICO AF study.

To relate clinical characteristics of SCAF with biochemical changes, additional biomarkers will be sampled in the blood of patients included in this study. Specifically, analysis of biomarkers associated with myocardial fibrosis, inflammation, oxidative stress and heart failure will be assessed. In a prespecified sub-analysis, blood, ECG and ICD data in combination with the actual documentation of SCAF will be used to evaluate treatment decisions, and attempts will be made to construct a decision model guiding such treatment decisions.

### Patient selection

Patients with a class IA indication for ICD therapy as primary prevention for SCD and age between 65 and 80 years may be eligible for this study. Main exclusion criteria are current AF or a history of AF and the use of vitamin K antagonists or non-vitamin K antagonists (NOACs). Complete inclusion and exclusion criteria are shown in Tab. 1.

**Table 1:** Inclusion and exclusion criteria

Inclusion	Exclusion
Age 65 ≥ 80 years	Current AF or a history of AF
CAD	Use of vitamin K antagonist or NOACs
LVEF ≤35%	Use of class I or III antiarrhythmic drugs
Life expectancy >2 years	Prosthetic heart valves
	Dilated or hypertrophic cardiomyopathy
	Congenital heart disease for which surgical correction was performed
	Inherited arrhythmia syndrome
	Active malignant disease
	A history of anthracycline use
	A history of TIA, stroke or systemic embolism
	Life expectancy <2 years

*CAD* coronary artery disease, *LVEF* left ventricular ejection fraction, *AF* atrial fibrillation, *NOAC* non-vitamin K oral anticoagulant, *TIA* transient ischaemic attack

### Device implantation and programming

**Fig. 2:**
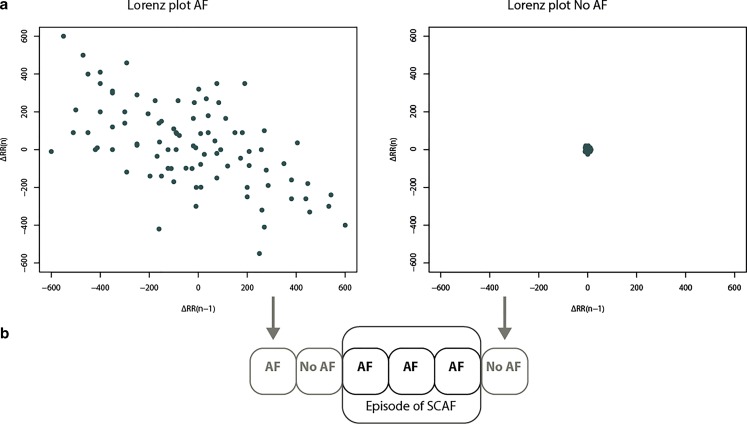
**a** Lorenz plot of ∆RR intervals (−600 ms to +600 ms) during a 2-min block. *Left:* the dispersion of *dots* during a 2-min block labelled as AF by the device. *Right:* the dispersion of *dots* during a 2-min block labelled as NO AF by the device. **b** An episode is detected and stored as SCAF if there are three consecutive 2 min-blocks labelled as AF. *AF* atrial fobrillation, *SCAF* subclinical atrial fibrillation

Included patients will receive a single chamber ICD (VISIA AF™, Medtronic) with an integrated AF detection algorithm as part of standard of care ICD therapy. The VISIA AF™ is CE marked and commercially available in the Netherlands. It enables continuous monitoring for SCAF in single chamber ICD patients, by detecting RR interval based SCAF episodes of ≥6 min [6]. Episodes will be classified as SCAF at the end of the third 2‑min period of SCAF (Fig. 2b). The label of each 2‑min block is the result of a newly created Lorentz plot: a scatterplot of RR interval versus the preceding RR interval (Fig. 2a). As a standard setting, the ICD will only store the cumulative duration and the number of separate SCAF episodes. However, the ICD contains a dedicated Holter that communicates with the ICD and allows recording of the duration of individual AF episodes, thereby providing insight into the temporary distribution of AF episodes. The Holter monitor will only be enabled in patients in whom SCAF is detected during the study. All patients will be equipped with a remote monitoring system.

### Follow-up

The occurrence of SCAF will be monitored via remote monitoring on a monthly basis as described previously [8, 9], for 1 year or until 20% of patients have had a first SCAF episode. All subjects will be followed at least until the last subject completes their 12-month follow-up visit. Patients will be seen at the outpatient clinic at 6 and 12 months for repeated blood testing and ECG.

### Sample size and power calculations

A total of 50 patients will be included. This sample size is powered for the primary endpoint, namely the incidence of SCAF in patients with CAD and a primary indication for a single chamber ICD. As this is an exploratory analysis, and data on the incidence of SCAF in patients with CAD and a reduced LVEF are inconclusive, we powered the study on an estimated incidence of AF of 8.3% in patients with CAD and a reduced LVEF without the use of a cardiovascular implantable electronic device (CIED) to detect AF, and an expected event rate of 20% of the primary endpoint detected by a CIED in a high-risk population [5, 10, 11]. Using a one-group, one-sided χ^2^ test, with a significance level of 0.05 alpha and 80% power, 46 patients would be required. Taking into consideration a loss of follow-up of approximately 10%, this would result in a total number of 50 subjects to be included. If the power is not achieved after 1 year, patients will be followed on an event-driven basis until 20% of patients demonstrate SCAF.

## Discussion

Timely detection of AF is of high clinical importance in patients at a high risk of stroke, who will benefit most from anticoagulation therapy. In addition, detection of AF in ICD patients may prevent worsening of heart failure and the occurrence of inappropriate shocks. Dual and triple chamber ICDs and recently single chamber ICDs are able to detect SCAF by detecting atrial high rates (AHRE) from the atrial electrogram or by RR interval based algorithms. A similar algorithm is in use in the subcutaneous ICD. By estimating the incidence of SCAF in a high-risk population of patients with CAD and a reduced LVEF, the INDICO AF study will advance our understanding of the value of detection of SCAF and its consequences. Subsequently, the INDICO AF study may provide a clinical justification for active monitoring for new onset (subclinical) AF.

### Clinical implication of device-detected AF

Of all strokes, 25% are of unknown cause and undiagnosed AF may be the aetiological factor [12]. Continuous monitoring of AF is superior to conventional follow-up visits or intermittent rhythm monitoring to identify AF after a cryptogenic stroke or detect AF recurrence after therapeutic interventions [9, 13]. In patients with a pacemaker, the incidence of SCAF is high and associated with an increased risk of stroke [14]. A relation between the duration of device-detected AF and stroke was demonstrated in a subanalysis of the ASSERT trial, although the event rate was lower than in patients with overt AF [15]. Not surprisingly, CIEDs with the ability to detect SCAF are of great interest to timely detect AF and prevent AF-related complications, as the population receiving ICD is generally older and has comorbidities increasing the risk for both AF and stroke. Indeed, the incidence of SCAF detected by an implantable loop recorder in patients without a history of AF but with a CHADS score ≥2 was 34% in 1 year [16].

Whether or not the implications with regard to oral anticoagulation are the same in patients with device-detected AHRE is subject of two ongoing clinical trials [17, 18]. There is no consensus either on whether AHRE detected from an atrial electrogram should be valued the same as SCAF detection based on irregularity of the RR intervals, or that stroke risk is merely driven by the duration of SCAF episodes [15]. Irrespective of whether anticoagulation should be started in all patients, establishing the diagnosis of AF is critical to determine increased stroke risk. This also applies for patients with an embolic stroke of undetermined source, as it was shown that starting rivaroxaban without documentation of AF in those patients was found to be futile compared with standard of care with aspirin [19].

CIEDs with an atrial lead, such as conventional dual and triple chamber devices, detect SCAF by recording episodes of AHRE in combination with or without the use of pre-programmed algorithms. The incidence of AHRE is high in patients with CIEDs, and CIED-detected AHRE lasting longer than 6 min is indeed associated with an increased risk of stroke [20, 21]. However, it is worth noting that not all AHRE equal atrial arrhythmias or atrial fibrillation. In addition, the high rate of SCAF in patients detected by AHRE may be overestimated as the atrial lead needed for the detection of AHRE may cause transient atrial pro-arrhythmia [22].

CIEDs without an atrial electrode such as implantable loop recorders, the VISIA AF™ and S‑ICD mostly use RR interval based algorithms for the detection of SCAF [6, 12, 23]. The AF interval-based algorithm in the VISIA AF™ ICD is based on the same RR interval detection and P wave evidence algorithms that are integrated in implantable loop recorders for the detection of AF (e. g. Reveal XT, Reveal Linq) [7, 12]. The VISIA AF™ uses an intracardiac ventricular ECG instead of a subcutaneous ECG, and episodes of SCAF are defined as the result of three 2‑min blocks of AF instead of one (Fig. 2). These extensions have resulted in an increased positive predictive value compared with strategies used in implantable loop recorders. Contrary to AF detection using atrial electrograms, the RR interval based algorithm is AF specific, and will thereby not identify atrial flutter or atrial tachycardia. High-risk patients with RR interval based implantable loop recorders show a substantial incidence of SCAF [10], but data on the clinical importance of SCAF detected by single chamber ICDs are inconclusive. The INDICO AF study will not only investigate the incidence SCAF in high-risk patients with a single chamber ICD, but will also determine the burden of SCAF. The combination of these device-detected SCAF episodes and the documentation of clinical characteristics, clinical follow-up and plasma biomarkers for fibrosis, inflammation, oxidative stress and heart failure will be introduced in a decision model that may allow preterm identification of those patients who will develop AF or AF-related complications.

### Limitations of device-detected SCAF

Advances in CIED technology have made it possible to continuously monitor SCAF in different patient populations, leading to an increasing number of patients diagnosed with SCAF. SCAF is associated with an increased risk of stroke; however, there are no guidelines on oral anticoagulant (OAC) therapy yet as data on the benefit of OAC in this population is lacking. Ongoing trials will evaluate the value of OAC in patients with detected devices [17, 18]. A subanalysis from the ASSERT trial showed that only patients with SCAF duration longer than 24 h significantly increase the risk of stroke [15]. It is therefore debatable if—and if so when—SCAF truly represents clinical AF, and whether SCAF only mirrors the high-risk population. Future studies are needed to standardise the definition of SCAF and the considerations in therapy.

## Summary

The INDICO AF study will evaluate the incidence and burden of SCAF in high-risk patients with a single chamber ICD, to gain insight into the value of detected SCAF by the use of an integrated AF detection algorithm and remote monitoring. With a small sample size of only 50 patients, it is important to note that the INDICO AF study will be an observational study, intending to serve as a pilot study for upcoming large international trials on AF detection algorithms in single chamber ICD patients.
